# The Effects of Pharmacological Compounds on Beat Rate Variations in Human Long QT-Syndrome Cardiomyocytes

**DOI:** 10.1007/s12015-016-9686-0

**Published:** 2016-09-19

**Authors:** Jukka Kuusela, Jiyeong Kim, Esa Räsänen, Katriina Aalto-Setälä

**Affiliations:** 1 Institute of Biomedical Technology, University of Tampere, Tampere, Finland; 2 BioMediTech, Tampere, Finland; 3 Department of Physics, Tampere University of Technology, Tampere, Finland; 4 School of Medicine, University of Tampere and Tampere University Hospital, Finn-Medi 5, Biokatu 12, FI-33014 Tampere, Finland; 5 Heart Hospital, Tampere University Hospital, Tampere, Finland

**Keywords:** Induced pluripotent stem cell, Long QT syndrome, Cardiomyocytes, Multielectrode array, Detrended fluctuation analysis, Fractals, Nonlinear dynamics

## Abstract

Healthy human heart rate fluctuates overtime showing long-range fractal correlations. In contrast, various cardiac diseases and normal aging show the breakdown of fractal complexity. Recently, it was shown that human induced pluripotent stem cell-derived cardiomyocytes (hiPSC-CMs) intrinsically exhibit fractal behavior as in humans. Here, we investigated the fractal complexity of hiPSC-derived long QT-cardiomyocytes (LQT-CMs). We recorded extracellular field potentials from hiPSC-CMs at baseline and under the effect of various compounds including β-blocker bisoprolol, ML277, a specific and potent I_Ks_ current activator, as well as JNJ303, a specific I_Ks_ blocker. From the peak-to-peak-intervals, we determined the long-range fractal correlations by using detrended fluctuation analysis. Electrophysiologically, the baseline corrected field potential durations (cFPDs) were more prolonged in LQT-CMs than in wildtype (WT)-CMs. Bisoprolol did not have significant effects to the cFPD in any CMs. ML277 shortened cFPD in a dose-dependent fashion by 11 % and 5–11 % in WT- and LQT-CMs, respectively. JNJ303 prolonged cFPD in a dose-dependent fashion by 22 % and 7–13 % in WT- and LQT-CMs, respectively. At baseline, all CMs showed fractal correlations as determined by short-term scaling exponent α. However, in all CMs, the α was increased when pharmacological compounds were applied indicating of breakdown of fractal complexity. These findings suggest that the intrinsic mechanisms contributing to the fractal complexity are not altered in LQT-CMs. The modulation of I_Ks_ channel and β1-adrenoreceptors by pharmacological compounds may affect the fractal complexity of the hiPSC-CMs.

**Electronic supplementary material:**

The online version of this article (doi:10.1007/s12015-016-9686-0) contains supplementary material, which is available to authorized users.

## Introduction

Heart rate dynamics have been previously analyzed using conventional linear and newer nonlinear methods in healthy and diseased states (for review see Perkiömäki [1]). The nonlinear indices of the RR variations include fractals, which are geometrically defined as objects composed of subunits (and sub-subunits) that sustain self-similarity on different measurement scales [2]. The characteristic feature of fractals is 1/f-like fluctuations, which has been shown to be present in a healthy human heartbeat [3–6]. Such fluctuations possess long-range correlations indicative of a memory effect, which means that the heart rate is not only related to immediately preceding value but also to values in the remote past [2].

In the heart rate time series, a breakdown of 1/f-like fluctuations may lead into either completely uncorrelated randomness (white noise) or into total correlation resembling random walk (Brownian motion). A number of studies have shown that in various cardiac disease states (e.g. congestive heart failure, myocardial infarction) the fractal 1/f-like long-range correlations of the heartbeat breakdown producing more uncorrelated randomness [7–13]. Furthermore, normal aging has been associated with the breakdown of fractal complexity producing more total correlation in the heartbeat dynamics [6, 14, 15]. Thus, it is thought that the breakdown of fractal complexity in heart rate dynamics may cause the system to be less adaptable and less responsive to unpredictable stimuli and stresses increasing susceptibility to injury and illness [6, 16].

The biological origin of the fractal-like behavior has not been yet fully established and is somewhat contradictory. In the heart, the origin of the fractal-like behavior is thought to result from complex interaction between vagal and sympathetic inputs of the autonomous nervous system [17, 18]. Experimental observations in humans have supported this notion [19–23], although opposing views have been presented [24]. However, evidence suggests that monolayer cultures of rat ventricular cardiomyocytes and human induced pluripotent stem cell-derived cardiomyocytes (hiPSC-CMs) lacking autonomic control exhibit fractal-like complexity, which indicates that the autonomous nervous system input is not necessary for fractal dynamics [25, 26]. It has been further demonstrated that single cardiomyocytes also exhibit fractal-like complexity and that intracellular Ca^2+^-cycling mechanisms contribute to the fractal complexity of the single cardiomyocytes [27].

Long QT syndrome (LQTS) is a potentially severe life-threatening arrhythmic cardiac disease characterized by prolonged QT interval on the electrocardiogram. LQTS is associated with torsades de pointes, a special type of ventricular tachycardia, which may degenerate into ventricular fibrillation and cause sudden cardiac death [28]. Inherited forms of LQTS are a result of mutations in the cardiac ion channel coding genes. One of the most common LQTS genes is *KCNQ1*, which encodes the α-subunit of the voltage-gated potassium channel responsible for the slow delayed rectifier K^+^ current (I_Ks_) [29, 30]. In Finland, the prevalence of gene mutations associated with LQTS is high (0.4 % of Finnish population), which has been explained to be caused by four founder mutations with one of them being C-terminal G589D missense mutation in *KCNQ1* gene [31, 32]. The hiPSCs represent excellent research tool to study the pathophysiology of inherited cardiac diseases [33–41]. Until now, the fractal dynamics of hiPSC-LQT-CMs have not been investigated.

Here, we utilize patient-specific LQTS disease model [41] to study the fractal dynamics in the symptomatic and asymptomatic LQTS type 1 (LQT1)-specific CMs carrying Finnish founder mutation G589D. The aim of this study was to investigate the fractal dynamics of LQT-CMs in unmedicated and medicated conditions as compared to healthy control and under the effect of various compounds affecting cardiac action potential.

## Material & Methods

### ECG Recordings and Human Induced Pluripotent Stem Cell Generation

The study was approved by the ethical committee of Pirkanmaa Hospital District (R08070). Participants who volunteered for the study gave their consent. The ECGs were recorded using MARS-Holter from a healthy individual, asymptomatic LQT-mutation carrier and symptomatic LQT-patient. The LQT-patients are on bisoprolol medication. The healthy individual has no medication. Human iPSCs were generated as described earlier [42]. The LQT1-specific hiPSCs were derived from patients’ skin fibroblasts carrying G589D missense mutation in *KCNQ1* [41, 43].

### Patient Characteristics

Skin biopsies with LQT1 mutation were obtained from a symptomatic 41-year old female patient (QTc interval, 456 ms) and from an asymptomatic 28-year old female mutation carrier (QTc interval, 428 ms). Both carry the *KCNQ1* G589D mutation. The symptomatic 41-year old patient had experienced seizures, episodes of unconsciousness and syncope before β-blocker (bisoprolol) medication. The healthy control human iPS cells were derived from skin fibroblasts of a healthy 55-year old female (QTc interval, 406 ms) [44].

### Human Induced Pluripotent Stem Cell Culture, Differentiation and Characterization

Human iPS cells were cultured and differentiated as previously described [43]. All the hiPSC lines (UTA.04602.WT, UTA.00208.LQT1, UTA.00211.LQT1, UTA.00303.LQT1 and UTA.00313.LQT1) and the differentiated CMs from them have been previously characterized elsewhere [41, 43, 44].

### Multielectrode Array Recordings and Data Analysis

In this study, 30–45 days old hiPSC-CMs were used for the experiments. Spontaneously beating cardiomyocyte clusters were manually dissected and plated on 6-well MEAs (6-well MEA 200/30iR-Ti-tcr, Multichannel Systems, Reutlingen, Germany), which were first coated with fetal bovine serum (FBS, Invitrogen) for 30 min at room temperature and then with 0.1 % gelatine (Sigma Aldrich) for 1 h at room temperature. The cardiomyocyte clusters were cultured in EB-medium: KO-DMEM with 20 % FBS, NEAA, Glutamax and penicillin/streptomycin. The experiments were conducted in 5 % FBS containing EB-medium (5 % EB-medium). Before drug tests, the field potentials originating from the spontaneously beating cardiomyocytes were recorded for 30 min (baseline) at +37 °C with the MEA platform (MEA2100-2 × 60–2, Multichannel Systems, Reutlingen, Germany) using 10 kHz sampling frequency and MC_Rack (Multichannel Systems, Reutlingen, Germany) software. After the 30-min baseline measurement, the MEA plate was put on +37 °C thermal plate (Tokai Hit, Japan) for keeping the temperature stable while adding drugs. The following drugs were used in the study: Bisoprolol (Sigma-Aldrich), ML277 (Tocris Bioscience) and JNJ303 (Tocris Bioscience). The drugs were dissolved in dimethyl sulfoxide (DMSO, Sigma-Aldrich) according to manufacturer’s instructions. The bisoprolol concentrations were chosen based on its therapeutic blood serum concentration range [45]. For bisoprolol, 260 nM (upper limit of the therapeutic serum concentration) and 520 nM (twice the upper limit of the therapeutic concentration) concentrations were used. ML277 (I_Ks_ channel activator) concentrations of 1 μM and 2 μM were chosen based on previous reports [46, 47]. The concentrations of I_Ks_ blocker JNJ303 (300 nM and 1000 nM) were chosen based on our previous study [41]. After drug addition, the MEA plates were incubated for 5 min at +37 °C thermal plate before the 30-min measurement (first drug concentration). After this, we added more drugs to the cells (second drug concentration) and similarly as before, recorded the field potentials for 30 min. We also conducted vehicle control experiments with similar protocol as described above, with the exception that no drugs but only DMSO (0.1 %) was added to the cells. The recording time for baseline and for each drug concentration was 30 min. The data obtained from MEA was analyzed by our in-house developed CardioMDA software, which averages field potential signals using cross correlation algorithms [48]. From each recording, the last 2 min from the 30-min recording were chosen for averaging the field potential signals. For determining the field potential duration (FPD), the onset was determined as the beginning of depolarizing peak and the offset as T_max_ of the repolarizing wave. The Bazett’s and Fridericia’s formula were used to calculate the corrected field potential duration (cFPD).

### Detrended Fluctuation Analysis

We applied detrended fluctuation analysis (DFA) to the RR-intervals. DFA is one of the most used time-series analysis methods that gives a reliable estimate for the existence and the characteristics of long-range correlations in the data [7]. DFA has been applied in various fields of science ranging from physiological signals such as heartbeat and gait [49] to, for example, musical rhythms [50, 51], rainfall statistics [52], structural properties of DNA [53], and electronic quantum transport [54].

A detailed description of DFA can be found in the above-listed references and here we only summarize the main steps. First, we take the peak-to-peak intervals of the hiPSC-CM or ECG data set and subtract the mean value, so that we consider the *fluctuations* around the mean. Next, we integrate the series by taking a cumulative sum of the fluctuations. The time axis is then divided into non-overlapping windows, and in each window, a least-squares line (trend) is fit to the data. The root-mean-square deviations from the trend (residuals) are averaged through the whole data set. This procedure is repeated for different window sizes. As a result, we get relationship between the window size and the average fluctuation (within that window size). The slope in this plot in a log-log scale corresponds to the DFA exponent α. White noise with no correlation between consecutive values has α = 0.5, whereas Brownian motion with strongly correlated values generated by uncorrelated consecutive *increments* has α = 1.5. In general, intermediate predictability between these limits with 0.5 < α ≤ 1.5 indicates long-range (fractal) correlations. Anti-correlations are characterized by −0.5 < α < 0.5. The special case of pink noise α = 1 corresponds to 1/f behavior.

### Statistical Analyses

From MEA data, one-way ANOVA followed by Dunnet’s post hoc test was performed to test differences in baseline values between control and LQT cell lines (IBM version 22.0; SPSS Inc., Chicago, USA). If datasets did not meet ANOVA requirements (normal distribution, equal variances), nonparametric test Mann-Whitney U followed by alpha correction was employed to compare control cell line and LQT cell lines. In drug experiments, the baseline and the effect of drug concentrations were compared using paired sample t-test. Similarly, if datasets did not meet the requirements for t-test, nonparametric Wilcoxon test was employed. From DFA data, paired sample t-test was employed to determine the statistical differences between baseline and each drug concentration. The *p* < 0.05 was considered statistically significant. The levels of significance are represented as (*) p < 0.05, (**) *p* < 0.01 and (***) *p* < 0.001. The cFPD prolongations of >10 % are considered physiologically significant. The data is presented as mean ± standard deviation (SD).

## Results

### The Effect of Pharmacological Compounds to the hiPSC-CM Clusters

**Fig. 1:**
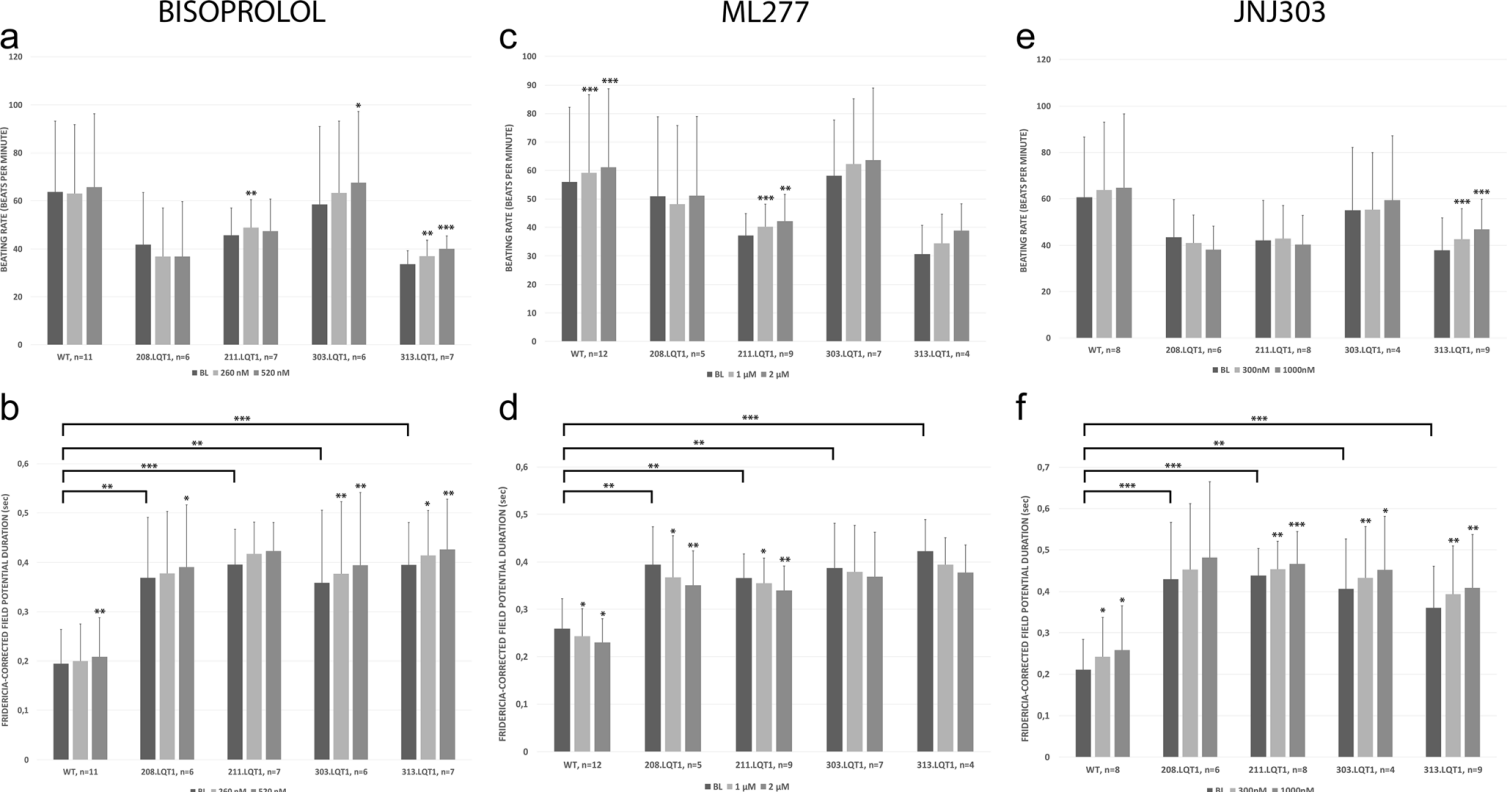
The effects of various compounds to the human induced pluripotent stem cell (hiPSC)-derived cardiomyocytes’ field potential parameters. The upper row depicts the beating rate (BR) and the lower row Fridericia-corrected field potential duration (cFPD). The baseline cFPDs of long QT-specific cardiomyocytes were significantly more prolonged than in healthy wild type-cardiomyocytes. The asterisks on top of the bars depict the statistical significance for mean BR or cFPD change compared to baseline values. Significance levels are indicated by (*) *p* < 0.05, (**) *p* < 0.01 and (***) *p* < 0.001, respectively

The 208.LQT1 and 211.LQT1 were derived from symptomatic patient whereas 303.LQT1 and 313.LQT1 were derived from asymptomatic mutation carrier. The baseline cFPDs (Fridericia’s correction) were significantly more prolonged in hiPSC-LQT-CMs than in healthy WT-CMs (Fig. 1b, d, f). Similar results were obtained when Bazett’s FPD correction was used (Supplemental Fig. 1). In contrast, we did not find any significant differences in the beating rates (BRs) between WT- and LQT-CMs (Fig. 1 a, c, e).

#### β-Blocker Bisoprolol

**Fig. 2:**
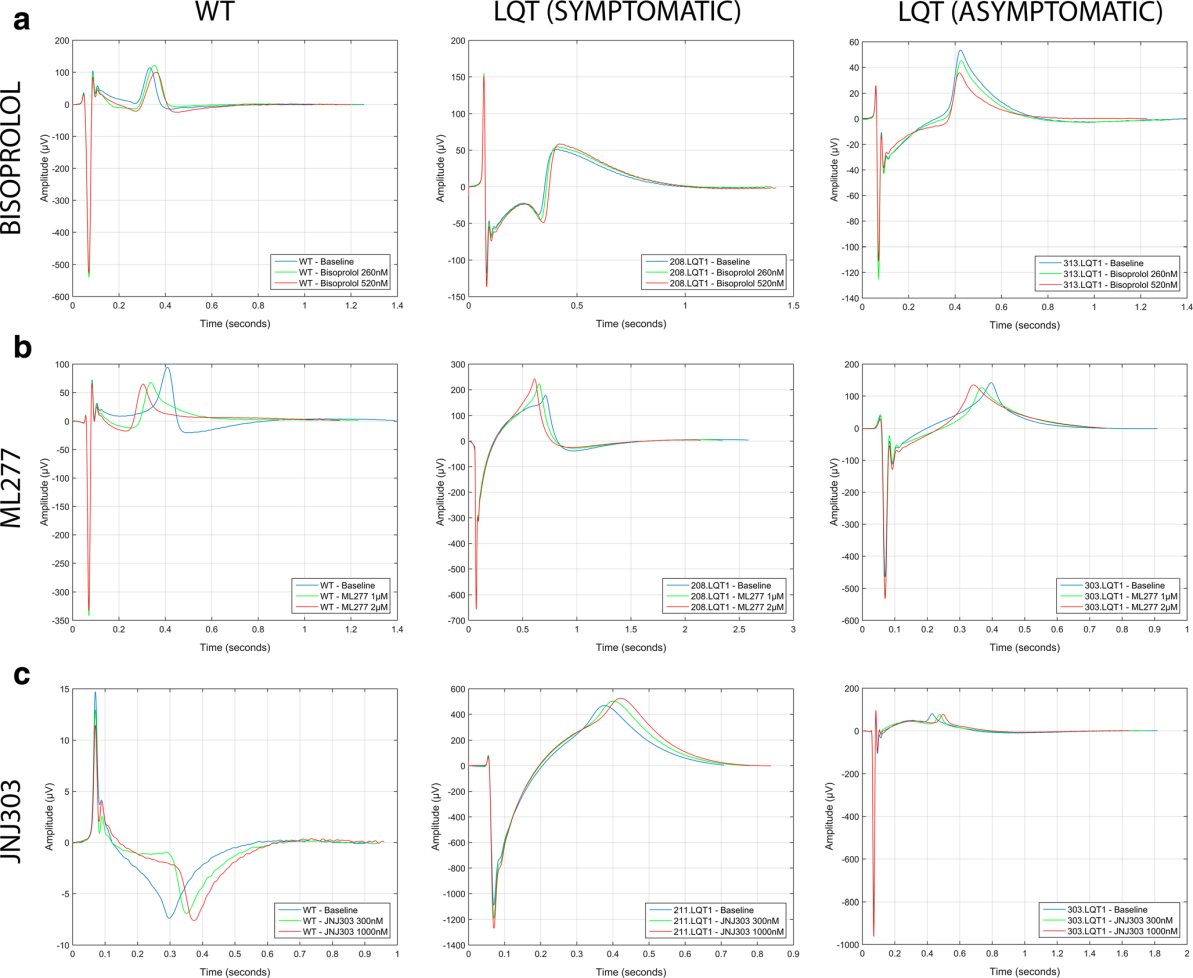
The representative traces of the human induced pluripotent stem cell (hiPSC)-derived wild type- and long QT (LQT)-specific cardiomyocytes under the effect of various compounds. The LQTs are grouped according to symptomatic (208.LQT1, 211.LQT1) and asymptomatic cases (303.LQT1, 313.LQT1) shown in the middle and in the right column, respectively. **a**) β-blocker Bisoprolol, **b**) I_Ks_ activator ML277 **c**) I_Ks_ blocker JNJ303. Notice, how the T_max_ of the repolarization wave is relatively unchanged between baseline and different bisoprolol concentrations depicting marginal effect of the β-blocker to the corrected field potential duration (cFPD). However, I_Ks_ activator ML277 shows clear shortening of cFPD seen by shift in the T_max_. Similarly, but conversely to ML277, I_Ks_ blocker JNJ303 shows clear prolongation of cFPD assessed by the shift in the T_max_

The bisoprolol did not cause any major differences in the BRs of the hiPSC-CMs (Fig. 1a). We noted a slightly increasing trend of BR with increasing bisoprolol concentration in the hiPSC-CMs from asymptomatic mutation carrier (15 % for 303.LQT1 and 19 % for 313.LQT1). As for the cFPD, bisoprolol caused only mild cFPD prolongation in the WT-CMs (3–7 %) at the concentration range of 260–520 nM. Similar cFPD prolongations were seen in LQT-CMs from symptomatic patient (2–6 % for 208.LQT1 and 5–7 % for 211.LQT1) and from asymptomatic mutation carrier (5–10 % for 303.LQT1 and 5–8 % for 313.LQT1) at the concentration range of 260-520 nM (Fig. 1b). The representative bisoprolol traces for WT- and LQT-CMs are illustrated in Fig. 2a.

#### I_Ks_ Activator ML277

The ML277 caused significant increase in the BRs of WT and 211.LQT1, 9 % and 14 %, respectively (Fig. 1c). Overall, the trend of increasing BR with increasing ML277 concentration was observed although statistical significance was not found for all the cell lines. As expected, the ML277 shortened the cFPD in a dose-dependent manner, although the cFPD shortening was relatively mild (Fig. 1d). In the WT, the shortening of cFPD was 11 % whereas in the LQTs it ranged from 5 to 11 % at the concentration range of 1-2 μM. The representative ML277 traces for WT- and LQT-CMs are illustrated in Fig. 2b.

#### I_Ks_ Blocker JNJ303

Blocking the I_Ks_ channel with JNJ303 did not significantly change the BRs of the WT- or LQT-CMs except in 313.LQT1, in which the 23 % increase in the BR was observed (Fig. 1e). JNJ303 showed a dose-dependent cFPD prolongation in the WT- and LQT-CMs (Fig. 1f). The most increment in cFPD was seen in the WT (22 %) whereas in the LQTs, the cFPD increase was around 7–13 % at the highest concentration (1000 nM). The representative JNJ303 traces for WT- and LQT-CMs are illustrated in Fig. 2c.

### Detrended Fluctuation Analysis (DFA) of Healthy Control- and LQT-Specific Cardiomyocytes and ECG Data

**Fig. 3:**
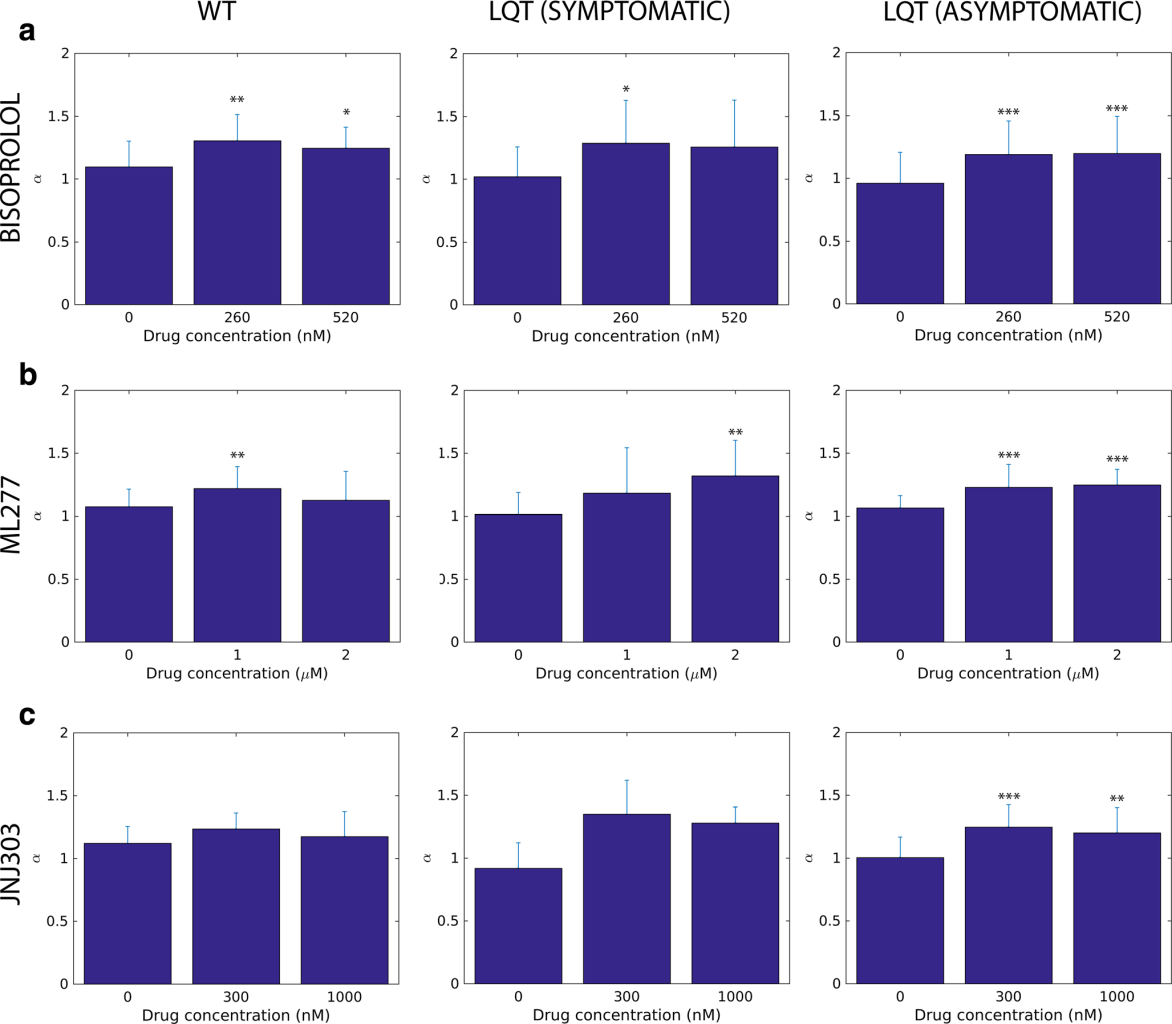
The detrended fluctuation analysis (DFA) α scaling exponents of the human induced pluripotent stem cell (hiPSC)-derived wild type- and long QT (LQT)-specific cardiomyocytes. The LQTs are grouped according to symptomatic (208.LQT1, 211.LQT1) and asymptomatic cases (303.LQT1, 313.LQT1) shown in the middle and in the right column, respectively. **a**) (row) β-blocker Bisoprolol, **b**) I_Ks_ activator ML277 **c**) I_Ks_ blocker JNJ303. At baseline, the α scaling exponents are close to 1 in all hiPSC-CMs. The addition of various compounds to the hiPSC-CMs increased the α scaling exponent closer to Brownian motion (α = 1.5). The asterisks on top of the bars depict the statistical significance for mean α scaling exponent change compared to baseline values. Significance levels are indicated by (*) p < 0.05, (**) p < 0.01 and (***) p < 0.001, respectively

We first determined the fractal scaling exponent α from human subjects who participated to the study (*n* = 3). Results are shown in Table 1. Next, we determined the scaling exponent α from hiPSC-CMs derived from the same human subjects participating to the study. In Fig. 3 we show the DFA results of the peak-to-peak fluctuations for healthy control- (WT) and LQT-specific CMs when exposed to bisoprolol (Fig. 3 a), ML277 (Fig. 3 b), or JNJ303 (Fig. 3c). The first bar in all the subplots corresponds to the baseline with zero drug concentration. The LQT data is grouped according to symptomatic (208.LQT1, 211.LQT1) and asymptomatic cases (303.LQT1, 313.LQT1) shown in the middle and in the right column, respectively.

**Table 1:** The patient characteristics and detrended fluctuation analysis from ECG data

	Healthy individual	LQT1-patient (symptomatic)	LQT1-patient (asymptomatic)
Age	55	41	28
Medication	-	Bisoprolol	Bisoprolol
QTc (ms)	406	456	428
α	1.21 ± 0.03	1.08 ± 0.02	1.07 ± 0.02

We found that all the average baseline values for the DFA scaling exponent are close to one, i.e., α ~ 0.9–1.1. There was no notable difference in the baseline α values between the WT and the LQTs. This is in line with our ECG data, which shows α of 1.08, 1.07 and 1.21 for symptomatic and asymptomatic LQT patient as well as for healthy individual, respectively (Table 1). However, all the pharmacological compounds lead to an increase in the DFA scaling exponent α toward Brownian motion (α = 1.5) when compared to the baseline (Fig. 3). The trend is visible both in the WT and in the LQTs. In most cases a further increase in the concentration does not affect α. When comparing all the α values of WT and LQTs, we did not find any statistical difference between the groups. We also conducted vehicle control experiments in which no pharmacological compounds but only DMSO (0.1 %) was added to the CMs. We did not observe any significant changes in the α scaling exponent during these recordings. The α was 1.024 ± 0.13 at baseline, 1.00 ± 0.12 (first DMSO addition) and 1.03 ± 0.13 (second DMSO addition) (*n* = 6). Thus, the change seen in α scaling exponent with various pharmacological compounds reflect the intrinsic properties of the pharmacological compounds themselves.

## Discussion

In human hearts, the fractal dynamics is thought to result from complex interaction between vagal and sympathetic inputs of autonomous nervous system [17, 18]. However, evidence suggest that the hiPSC-CMs also exhibit fractal-like complexity indicating intrinsic mechanisms of the CMs contributing to fractal dynamics [26, 27]. This study further supports the concept that healthy hiPSC-CMs lacking autonomous nervous system input exhibit fractal-like complexity at baseline condition. We also observed that the untreated (without any pharmacological intervention) hiPSC-LQT-CMs also appear to exhibit fractal-like complexity at baseline condition. This result is in line with the previous study in untreated patients with congenital LQTS [55]. Thus, our results further expand this concept suggesting that the intrinsic mechanisms contributing to the fractal-like complexity is not altered in hiPSC-LQT-CMs. However, it is of note that the results obtained were from 2 LQTS patients only and further studies on a larger population would be needed to definitely conclude the matter.

### The Electrophysiological Properties of the hiPSC-CMs

The baseline behavior of hiPSC-CMs is in line with our previous studies [41, 43]. Results obtained from this study clearly show that the cFPDs, corrected with either Bazett’s or Fridericia’s formulae, are more prolonged in LQT-CMs than in healthy WT-CMs. We also investigated the healthy and LQT-specific hiPSC-CMs under the effect of β-blocker bisoprolol and pharmacological compounds affecting specifically to cardiac ion channel I_Ks_. Overall, the drug effects in LQT-CMs appeared to be of similar magnitude to those of WT-CMs, similar to our previous findings [43].

### The Effect of Bisoprolol to the Fractal Complexity of hiPSC-CMs

The β-blockers are the standard and currently, the only treatment of choice for LQTS patients [56]. Here, in order for valid comparisons between cellular and whole heart data, we chose to study bisoprolol in hiPSC-CMs because the LQTS patients volunteered for this study were on bisoprolol medication. Bisoprolol, a β1-adrenoreceptor selective β-blocker, did not have physiological significance to the field potential parameters (>10 % cFPD prolongation) in any of the hiPSC-CMs at clinically relevant concentration. It is important to notice that we did not activate β-receptors with β-agonists prior to β-blocker application. However, the acute application of bisoprolol increased the α scaling exponent toward Brownian motion (α = 1.5) in all hiPSC-CMs at the upper limit of therapeutic concentration (260 nM). At 520 nM, the effect was attenuated. Although the baseline α data from hiPSC-LQT-CMs correlated with the ECG data (α ~ 1.0), bisoprolol α data did not correlate with the ECG data. The discrepancy between cellular and heart α data remains yet unclear but it may be attributed to e.g. acute application of the bisoprolol or in the absence of autonomous nervous system in hiPSC-LQT-CMs, which may affect to fractal complexity upon drug application. Previous human study has shown that the fractal complexity (α ~ 1.0) was unaltered in patients with congenital LQTS treated or untreated with β-blockers [55]. Thus, the β-blockers may not have significant effect on the α of the whole LQTS hearts. On the other hand, there is evidence from the human studies that the β-blocker treatment improves fractal dynamics of the heart in advanced congestive heart failure patients by increasing the α scaling exponent during 1–3-month therapy period [21–23]. It is completely unknown whether such differences result from the different disease states, the type of β-blocker used or treatment periods among other things. However, this study clearly shows that although the field potential parameters were not significantly changed, the intrinsic mechanisms contributing to fractal-like complexity were altered during acute β-blocker treatment. On the other hand, further studies would be needed to answer what the effect would be in long-term (chronic) situation and what the mechanism behind of this phenomenon is.

The significance of the fractal complexity resembling Brownian motion in the heart is unclear. The loss of fractality toward white noise (α = 0.5) has been found to predispose to severe life-threatening arrhythmias and cardiac death [9, 10, 13]. However, very little is known when the fractal dynamics of the heart become more correlated resembling Brownian motion (α = 1.5). Such phenomenon has been observed in healthy elderly subjects implying decrease of fractal complexity with age [6, 14, 15] and, although not yet proven, it has been suggested that such system would be more susceptible to injury and illness in the elderly [6, 16].

### The Effect of I_Ks_ Affecting Pharmacological Compounds to the Fractal Complexity of hiPSC-CMs

Next, we investigated the effects of the other two pharmacological compounds (ML277 and JNJ303) to hiPSC-CMs. The ion channel activator ML277 has been shown to augment specifically I_Ks_ current and shorten the action potential duration in both healthy and LQT-CMs [46, 47]. Similarly, in this study, the ML277 shortened the cFPD although the effect was relatively mild in these hiPSC-CM clusters at 1-2 μM concentration. Similarly, as was seen with bisoprolol, also ML277 resulted in the dysfunction of regulatory mechanisms contributing to fractal complexity as the α scaling exponent was increased toward Brownian motion in all hiPSC-CMs at 1 μM. However, at 2 μM a small difference was seen that in the WT-CMs α exponent decreased to baseline level whereas the LQT-CMs α exponents continued to increase from 1 μM, which was most prominently seen in CMs derived from symptomatic patient (208.LQT1 and 00,211.LQT1). Furthermore, the effect of JNJ303, a potent and specific I_Ks_ blocker [57] known to evoke torsades de pointes, was assessed in hiPSC-CMs. The WT-CMs appeared to be more sensitive to JNJ303 than LQT-CMs as measured by cFPD prolongation. This result is in line with our previous findings from single WT- and LQT-CMs measured with patch clamp using the same cell lines [41]. Although not yet proven in the experimental setting, this indicates that the I_Ks_ current is diminished in the LQT1-CMs harboring G589D missense mutation compared to healthy WT-CMs. Contradictory to our expectations, also the JNJ303 resulted in similar alterations of fractal complexity in hiPSC-CMs as ML277 by increasing the α scaling exponent. Taken together, these evidence imply that the modulation of the ion channel generating I_Ks_ current with pharmacological compounds may result in the dysfunction of the intrinsic mechanisms contributing to fractal complexity. Indeed, rhythmic ion channel activation and inactivation in pacemaker cells has been thought to contribute to the ultradian rhythmicity in addition to spontaneous Ca^2+^-cycling [58]. Previous study has shown that the disruption of intracellular Ca^2+^ handling causes alterations in the scaling exponent α, mostly by decreasing it [27]. Here, we have shown that at baseline, the rhythmicity of hiPSC-CMs is sustained as determined by long-range fractal correlations. Furthermore, the modulation of the ion channel generating I_Ks_ current with specific pharmacological compounds disrupts CM rhythmicity and fractal complexity.

### Potential Limitation of the Study

In this study, only the acute effects of the various compounds to the fractal-like complexity were assessed in hiPSC-CMs. Further studies would be needed to show the chronic, long-term effects of the compounds. Moreover, the mechanisms behind the alterations in fractal complexity were not studied. We did not investigate in detail the effect of β-adrenergic agonist to the long-range fractal correlations of hiPSC-CMs. Also, with this model we could not take into account the continuous exposure of sympathetic and parasympathetic stimuli affecting CMs in vivo. This study does not answer to what would be the implications of altered fractality toward Brownian motion in hiPSC-CMs in terms of long-term health, adaptability and responsiveness to unpredictable stimuli.

## Conclusions

In conclusion, the hiPSC-LQT-CMs appear to exhibit fractal-like complexity at baseline condition suggesting that the intrinsic mechanisms of LQT-CMs contributing to the fractal complexity are not altered. Although the effects of various compounds to the field potential parameters were as expected, the fractal-like complexity of the hiPSC-CMs was significantly altered in healthy as well as LQT-specific CMs. No significant differences in the α scaling exponent were found between WT- and LQT-CMs. These findings may suggest that the cardiac ion channel generating I_Ks_ current as well as the modulation of β1-adrenoreceptors by β-blocker bisoprolol may contribute to the fractal-like complexity of the hiPSC-CMs.

## Electronic supplementary material

ESM 1 The effects of various compounds to the human induced pluripotent stem cell-derived cardiomyocytes’ field potential parameters. The field potential durations (FPDs) were rate-corrected by Bazett’s formula. The baseline corrected FPDs (cFPDs) of long QT-specific cardiomyocytes were significantly more prolonged than in healthy wild type-cardiomyocytes. The results depicted here are similar to those of Fridericia-corrected FPD results. The asterisks on top of the bars depict the statistical significance for cFPD change compared to baseline values. Significance levels are indicated by (*) *p* < 0.05, (**) *p* < 0.01 and (***) *p* < 0.001, respectively. (GIF 990 kb)

High resolution image (TIFF 1222 kb)

## References

[1] Perkiömäki JS (2011). Heart rate variability and non-linear dynamics in risk stratification. Frontiers in Physiology.

[2] Goldberger AL (1996). Non-linear dynamics for clinicians: chaos theory, fractals, and complexity at the bedside. Lancet.

[3] Kobayashi M, Musha T (1982). 1/f Fluctuation of Heartbeat Period. I.E.E.E. Transactions on Bio-Medical Engineering.

[4] Peng C, Mietus J, Hausdorff JM, Havlin S, Stanley HE, Goldberger AL (1993). Long-range anticorrelations and non-Gaussian behavior of the heartbeat. Physical Review Letters.

[5] Pikkujämsä SM, Mäkikallio TH, Juhani Airaksinen KE, Huikuri HV (2001). Determinants and interindividual variation of R-R interval dynamics in healthy middle-aged subjects. American Journal of Physiology. Heart and Circulatory Physiology.

[6] Goldberger AL, Amaral LAN, Hausdorff JM, Ivanov PC, Peng C, Stanley HE (2002). Fractal dynamics in physiology: alterations with disease and aging. Proceedings of the National Academy of Sciences of the United States of America.

[7] Peng C, Havlin S, Stanley HE, Goldberger AL (1995). Quantification of scaling exponents and crossover phenomena in nonstationary heartbeat time series. Chaos.

[8] Ho KKL, Moody GB, Peng C (1997). Predicting survival in heart failure case and control subjects by use of fully automated methods for deriving nonlinear and conventional indices of heart rate dynamics. Circulation.

[9] Mäkikallio TH, Seppänen T, Airaksinen KEJ (1997). Dynamic analysis of heart rate may predict subsequent ventricular tachycardia after myocardial infarction. The American Journal of Cardiology.

[10] Mäkikallio TH, Koistinen J, Jordaens L (1999). Heart rate dynamics before spontaneous onset of ventricular fibrillation in patients with healed myocardial infarcts. The American Journal of Cardiology.

[11] Vikman S, Mäkikallio TH, Yli-Mäyry S (1999). Altered complexity and correlation properties of R-R interval dynamics before the spontaneous onset of paroxysmal atrial fibrillation. Circulation.

[12] Huikuri HV, Poutiainen A, Mäkikallio TH (1999). Dynamic behavior and autonomic regulation of ectopic atrial pacemakers. Circulation.

[13] Huikuri HV, Mäkikallio TH, Peng C, Goldberger AL, Hintze U, Møller M (2000). Fractal correlation properties of R-R interval dynamics and mortality in patients with depressed left ventricular function after an acute myocardial infarction. Circulation.

[14] Iyengar N, Peng C-, Morin R, Goldberger AL, Lipsitz LA. (1996). Age-related alterations in the fractal scaling of cardiac interbeat interval dynamics. American Journal of Physiology. Regulatory, Integrative and Comparative Physiology, 271(4 40–4):R1078–84. 10.1152/ajpregu.1996.271.4.R1078 8898003

[15] Pikkujämsä SM, Mäkikallio TH, Sourander LB (1999). Cardiac interbeat interval dynamics from childhood to senescence: comparison of conventional and new measures based on fractals and chaos theory. Circulation.

[16] Lipsitz LA, Goldberger AL (1992). Loss of ‘complexity’ and aging: potential applications of fractals and chaos theory to senescence. JAMA.

[17] Goldberger AL, West BJ (1987). Fractals in physiology and medicine. The Yale Journal of Biology and Medicine.

[18] West BJ, Goldberger AL (1987). Physiology in fractal dimensions. American Scientist.

[19] Tulppo MP, Mäkikallio TH, Seppänen T (2001). Effects of pharmacological adrenergic and vagal modulation on fractal heart rate dynamics. Clinical Physiology.

[20] Tulppo MP, Kiviniemi AM, Hautala AJ (2005). Physiological background of the loss of fractal heart rate dynamics. Circulation.

[21] Lin L, Lin J, C- D, Lai L, Tseng Y, Huang SKS (2001). Reversal of deteriorated fractal behavior of heart rate variability by beta-blocker therapy in patients with advanced congestive heart failure. Journal of Cardiovascular Electrophysiology.

[22] Ridha M, Makikallio TH, Lopera G (2002). Effects of carvedilol on heart rate dynamics in patients with congestive heart failure. Annals of Noninvasive Electrocardiology.

[23] Chiu K, Chan H, Chu S, Lin T (2007). Carvedilol can restore the multifractal properties of heart beat dynamics in patients with advanced congestive heart failure. Autonomic Neuroscience Basic Clinical.

[24] Tan CO, Cohen MA, Eckberg DL, Taylor JA (2009). Fractal properties of human heart period variability: physiological and methodological implications. The Journal of Physiology.

[25] Kucera JP, Heuschkel MO, Renaud P, Rohr S (2000). Power-law behavior of beat-rate variability in monolayer cultures of neonatal rat ventricular myocytes. Circulation Research.

[26] Mandel Y, Weissman A, Schick R (2012). Human embryonic and induced pluripotent stem cell-derived cardiomyocytes exhibit beat rate variability and power-law behavior. Circulation.

[27] Ben-Ari M, Schick R, Barad L (2014). From beat rate variability in induced pluripotent stem cell-derived pacemaker cells to heart rate variability in human subjects. Heart Rhythm.

[28] Schwartz PJ, Crotti L, Insolia R (2012). Long-QT syndrome from genetics to management. Circulation. Arrhythmia and Electrophysiology.

[29] Barhanin J, Lesage F, Guillemare E, Fink M, Lazdunski M, Romey G (1996). K(V)LQT1 and lsK (minK) proteins associate to form the I(Ks) cardiac potassium current. Nature.

[30] Sanguinetti MC, Curran ME, Zou A (1996). Coassembly of K(V)LQT1 and minK (IsK) proteins to form cardiac I(Ks) potassium channel. Nature.

[31] Marjamaa A, Salomaa V, Newton-Cheh C (2009). High prevalence of four long QT syndrome founder mutations in the Finnish population. Annals of Medicine.

[32] Piippo K, Swan H, Pasternack M (2001). A founder mutation of the potassium channel KCNQ1 in long QT syndrome: implications for estimation of disease prevalence and molecular diagnostics. Journal of the American College of Cardiology.

[33] Egashira T, Yuasa S, Suzuki T (2012). Disease characterization using LQTS-specific induced pluripotent stem cells. Cardiovascular Research.

[34] Bellin M, Casini S, Davis RP (2013). Isogenic human pluripotent stem cell pairs reveal the role of a KCNH2 mutation in long-QT syndrome. The EMBO Journal.

[35] Moretti A, Bellin M, Welling A (2010). Patient-specific induced pluripotent stem-cell models for long-QT syndrome. New England Journal of Medicine.

[36] Itzhaki I, Maizels L, Huber I (2011). Modelling the long QT syndrome with induced pluripotent stem cells.. Nature.

[37] Lahti AL, Kujala VJ, Chapman H (2012). Model for long QT syndrome type 2 using human iPS cells demonstrates arrhythmogenic characteristics in cell culture. Disease Models & Mechanisms.

[38] Matsa E, Rajamohan D, Dick E (2011). Drug evaluation in cardiomyocytes derived from human induced pluripotent stem cells carrying a long QT syndrome type 2 mutation. European Heart Journal.

[39] Ma D, Wei H, Zhao Y (2013). Modeling type 3 long QT syndrome with cardiomyocytes derived from patient-specific induced pluripotent stem cells. International Journal of Cardiology.

[40] Yazawa M, Hsueh B, Jia X (2011). Using induced pluripotent stem cells to investigate cardiac phenotypes in Timothy syndrome. Nature.

[41] Kiviaho AL, Ahola A, Larsson K (2015). Distinct electrophysiological and mechanical beating phenotypes of long QT syndrome type 1-specific cardiomyocytes carrying different mutations.. IJC Heart & Vasculature.

[42] Takahashi K, Tanabe K, Ohnuki M (2007). Induction of pluripotent stem cells from adult human fibroblasts by defined factors. Cell.

[43] Kuusela J, Kujala VJ, Kiviaho A (2016). Effects of cardioactive drugs on human induced pluripotent stem cell derived long QT syndrome cardiomyocytes. SpringerPlus.

[44] Ahola A, Kiviaho AL, Larsson K, Honkanen M, Aalto-Setälä K, Hyttinen J (2014). Video image-based analysis of single human induced pluripotent stem cell derived cardiomyocyte beating dynamics using digital image correlation. Biomedical Engineering Online.

[45] Schulz M, Schmoldt A (2003). Therapeutic and toxic blood concentrations of more than 800 drugs and other xenobiotics. Pharmacogenetics.

[46] Yu H, Lin Z, Mattmann ME (2013). Dynamic subunit stoichiometry confers a progressive continuum of pharmacological sensitivity by KCNQ potassium channels. Proceedings of the National Academy of Sciences of the United States of America.

[47] Ma D, Wei H, Lu J (2015). Characterization of a novel KCNQ1 mutation for type 1 long QT syndrome and assessment of the therapeutic potential of a novel IKs activator using patient-specific induced pluripotent stem cell-derived cardiomyocytes. Stem Cell Research & Therapy.

[48] Pradhapan P, Kuusela J, Viik J, Aalto-Setala K, Hyttinen J (2013). Cardiomyocyte MEA data analysis (CardioMDA)--a novel field potential data analysis software for pluripotent stem cell derived cardiomyocytes. PloS One.

[49] Peng, C., Hausdorff, J. M., & Goldberger, A. L. (1999). Fractal mechanisms in neural control: Human heartbeat and gait dynamics in health and disease. In J. Walleczek (Ed.), *Nonlinear Dynamics, Self-Organization, and Biomedicine*. Cambridge University Press.

[50] Räsänen E, Pulkkinen O, Virtanen T, Zollner M, Hennig H (2015). Fluctuations of hi-hat timing and dynamics in a virtuoso drum track of a popular music recording. PloS One.

[51] Hennig H, Fleischmann R, Fredebohm A (2011). The nature and perception of fluctuations in human musical rhythms. PloS One.

[52] Matsoukas C (2000). Detrended fluctuation analysis of rainfall and streamflow time series. Journal of Geophysical Research, D: Atmospheres.

[53] Peng C, Buldyrev SV, Havlin S, Simons M, Stanley HE, Goldberger AL (1994). Mosaic organization of DNA nucleotides. Physical Review E.

[54] Kotimäki, V., Räsänen, E., Hennig, H., & Heller, E. J. (2013). Fractal dynamics in chaotic quantum transport. *Physical Review E - Statistical, Nonlinear, and Soft Matter Physics, 88*(2). doi:10.1103/PhysRevE.88.022913. 10.1103/PhysRevE.88.022913 24032907

[55] Perkiömäki JS, Zareba W, Couderc J, Moss AJ (2001). Heart rate variability in patients with congenital long QT syndrome. Annals of Noninvasive Electrocardiology.

[56] Schwartz PJ, Ackerman MJ, George AL, Wilde AA (2013). Impact of genetics on the clinical management of channelopathies. Journal of the American College of Cardiology.

[57] Towart R, Linders JTM, Hermans AN (2009). Blockade of the IKs potassium channel: an overlooked cardiovascular liability in drug safety screening?. Journal of Pharmacological and Toxicological Methods.

[58] Yaniv Y, Lakatta EG (2015). The end effector of circadian heart rate variation: the sinoatrial node pacemaker cell. BMB Reports.

