# Supplementary figures and images for: The Effects of Pharmacological Compounds on Beat Rate Variations in Human Long QT-Syndrome Cardiomyocytes
Source: Stem Cell Rev. 2016 Sep 19;12(6):698–707. doi: 10.1007/s12015-016-9686-0 (PMC5106508; doi:10.1007/s12015-016-9686-0)

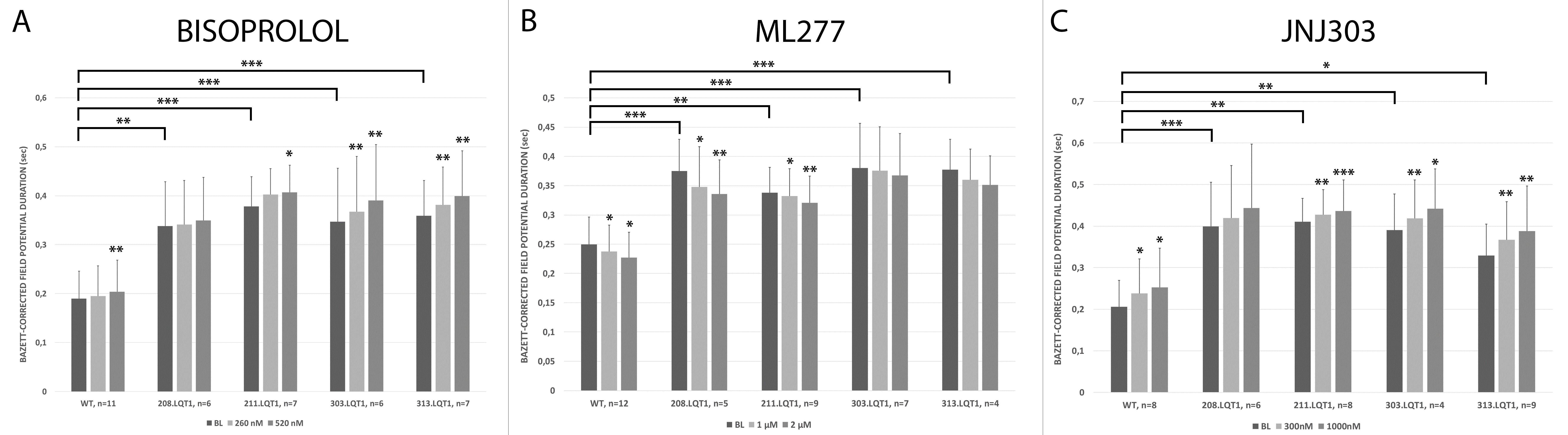

Supplement: Supplementary file 1 — The effects of various compounds to the human induced pluripotent stem cell-derived cardiomyocytes’ field potential parameters. The field potential durations (FPDs) were rate-corrected by Bazett’s formula. The baseline corrected FPDs (cFPDs) of long QT-specific cardiomyocytes were significantly more prolonged than in healthy wild type-cardiomyocytes. The results depicted here are similar to those of Fridericia-corrected FPD results. The asterisks on top of the bars depict the statistical significance for cFPD change compared to baseline values. Significance levels are indicated by (*) p < 0.05, (**) p < 0.01 and (***) p < 0.001, respectively. (GIF 990 kb) [file 12015_2016_9686_Fig4_ESM.gif]

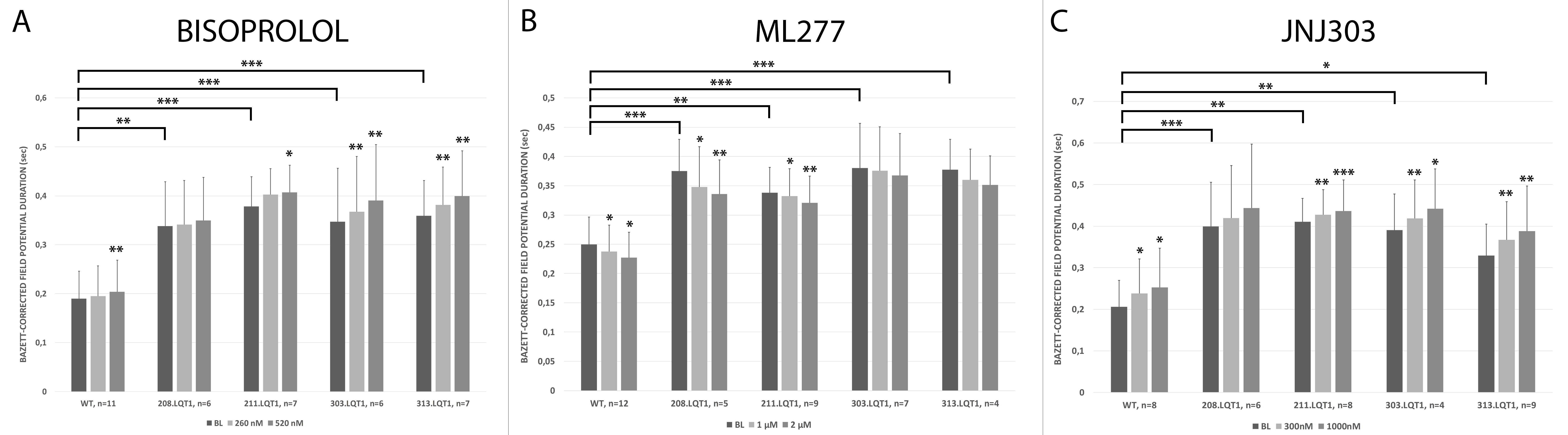

Supplement: Supplementary file 2 — High resolution image (TIFF 1222 kb) [file 12015_2016_9686_MOESM1_ESM.tif]
